# Mechanism of Ad5 Vaccine Immunity and Toxicity: Fiber Shaft Targeting of Dendritic Cells

**DOI:** 10.1371/journal.ppat.0030025

**Published:** 2007-02-23

**Authors:** Cheng Cheng, Jason G. D Gall, Wing-pui Kong, Rebecca L Sheets, Phillip L Gomez, C. Richter King, Gary J Nabel

**Affiliations:** 1 Vaccine Research Center, National Institute of Allergy and Infectious Diseases, National Institutes of Health, Bethesda, Maryland, United States of America; 2 GenVec, Incorporated, Gaithersburg, Maryland, United States of America; The Salk Institute for Biological Studies, United States of America

## Abstract

Recombinant adenoviral (rAd) vectors elicit potent cellular and humoral immune responses and show promise as vaccines for HIV-1, Ebola virus, tuberculosis, malaria, and other infections. These vectors are now widely used and have been generally well tolerated in vaccine and gene therapy clinical trials, with many thousands of people exposed. At the same time, dose-limiting adverse responses have been observed, including transient low-grade fevers and a prior human gene therapy fatality, after systemic high-dose recombinant adenovirus serotype 5 (rAd5) vector administration in a human gene therapy trial. The mechanism responsible for these effects is poorly understood. Here, we define the mechanism by which Ad5 targets immune cells that stimulate adaptive immunity. rAd5 tropism for dendritic cells (DCs) was independent of the coxsackievirus and adenovirus receptor (CAR), its primary receptor or the secondary integrin RGD receptor, and was mediated instead by a heparin-sensitive receptor recognized by a distinct segment of the Ad5 fiber, the shaft. rAd vectors with CAR and RGD mutations did not infect a variety of epithelial and fibroblast cell types but retained their ability to transfect several DC types and stimulated adaptive immune responses in mice. Notably, the pyrogenic response to the administration of rAd5 also localized to the shaft region, suggesting that this interaction elicits both protective immunity and vector-induced fevers. The ability of replication-defective rAd5 viruses to elicit potent immune responses is mediated by a heparin-sensitive receptor that interacts with the Ad5 fiber shaft. Mutant CAR and RGD rAd vectors target several DC and mononuclear subsets and induce both adaptive immunity and toxicity. Understanding of these interactions facilitates the development of vectors that target DCs through alternative receptors that can improve safety while retaining the immunogenicity of rAd vaccines.

## Author Summary

Recombinant adenovirus (rAd) vectors are remarkable for their ability to stimulate potent immune responses and to mediate highly efficient gene transfer. These vectors have been used extensively in human studies with generally acceptable tolerability. As with many bioactive compounds, adenoviruses can also cause potentially serious side effects, as observed in a human gene therapy trial several years ago that led to a fatality. The first manifestation of this toxicity is fever, but the relation of this side effect to the ability of the vector to stimulate immunity was unknown. We show that targeting of rAd vectors induces vaccine responses and toxicity through a previously unrecognized mechanism related to its attachment and entry into cells. We find that both adaptive immunity and fever are mediated by targeting of the rAd vector to dendritic cells and some monocytes, independent of the coxsackievirus and adenovirus receptor and RGD binding domains, mediated instead by the fiber shaft. This finding suggests that a distinct receptor present on dendritic and mononuclear cells mediates both effects. The immunogenicity of rAd vectors is dependent on targeting of virus through a specific fiber region and mediates rAd toxicity, which has implications for vaccine and gene therapy vector design that may help to improve rAd safety and efficacy.

## Introduction

The efficacy of adenovirus vectors as vaccines in many animal models of infectious diseases [1–3] and their immunogenicity in early clinical evaluation indicate their potential for human use. The mechanism underlying their strong immunogenicity and their relationship to adverse responses has not been well defined, nor has the connection between immunogenicity and adverse responses [4–6]. To address this issue, we evaluated the contribution of known receptor binding domains in the recombinant adenovirus serotype 5 (rAd5) fiber and penton base. The primary receptor recognition sequence resides in the knob region of the fiber. This domain has been localized to regions identified structurally [7], specifically in the AB loop, the B β-sheet, and the DE loop of the knob, and interacts with coxsackievirus and adenovirus receptor (CAR) [8–10]. Binding and internalization are facilitated through an interaction of an RGD motif in the penton base with integrin receptors [11,12]. To evaluate the contributions of these regions to targeting of rAd vectors to different cell types, we prepared vectors with mutations in these domains. Previous studies have shown that mutations in the CAR-binding domain inhibit infection of many cell types [10,13], and modifications of the RGD domain also affect targeting. Investigations of cell culture transduction do not always predict transduction of similar cell types in vivo. However, analysis of tissue transduction following intramuscular injection shows that elimination of both CAR and integrin receptor interactions greatly diminishes local transduction in muscle [14]. On the other hand, the long shaft in the fiber of Ad5 determines its hepatic tropism for systemic administration in mice [15,16]. In this study, we systematically investigated the contribution of these domains to the immunogenicity of Ad5-based vaccine vector when administered intramuscularly in mice and defined the molecular basis of its toxicity in a rabbit pyrogenicity model.

## Methods

### Preparation of rAd Vectors

The construction and propagation of the rAd5 vectors with wild-type (WT) capsid proteins and with mutated CAR- and integrin-binding motifs were previously described [14]. Total particle unit (PU) titer was determined by absorbance [17]. The chimeric rAd5+Ad35knob vector was a kind gift of Andre Leiber and is E1 and E3 deleted with the green fluorescent protein (GFP) expression cassette located in the E3 region [18].

### Isolation and Culture of Different Dendritic Cells from Peripheral Blood, Bone Marrow, and Spleen

Human peripheral blood was obtained from the National Institutes of Health Clinical Center Blood Bank (Bethesda, Maryland, United States), and mononuclear cells (PBMCs) were isolated by gradient centrifugation with Ficoll-Paque PLUS (Amersham Biosciences, http://www.amersham.com) as buffy coat and cultured in 10% RPMI medium (Invitrogen, http://www.invitrogen.com). Plasmacytoid dendritic cells (DCs) were isolated by magnetic cell sorting with BDCA-4 cell isolation kit (Miltenyi Biotec, http://www.miltenyibiotec.com).

Bone marrow (BM)-derived DCs were obtained from BM of BALB/c mice and cultured according to published methods [19]. More than 80% of these cells cultured with mGM-CSF after 1 wk expressed DC surface markers CD11b and CD11c as measured by flow cytometry.

Lymphoid DCs (CD8^+^ DCs) and plasmacytoid DCs (B220^+^ DCs) were isolated from mouse spleens by magnetic cell sorting according to the manufacturer's protocol (Miltenyi Biotec). More than 90% of these purified cells expressed CD8 or B220 as measured by antibody staining of the cells.

### Vaccination

Six- to 8-wk-old BALB/c female mice were used for immunogenicity studies. Mice were injected once with 100 μl of the specified rAd vectors encoding GFP or HIV Env (gp140ΔCFI) as the control vector at the indicated particle concentration bilaterally in the muscle with the use of needle and syringe. For each vector and dose, a group of five mice was injected with vector in PBS. All animal experiments were reviewed and approved by the Animal Care and Use Committee, Vaccine Research Center (VRC), National Institute of Allergy and Infectious Diseases (http://www.niaid.nih.gov/vrc) and performed in accordance with all relevant federal and National Institutes of Health guidelines and regulations.

### Cellular Immune Analysis

Three weeks after vaccination, mouse spleens were removed aseptically, gently homogenized to a single-cell suspension, washed, and resuspended to a final concentration of 10^6^ cells/ml. Harvested spleen cells (10^6^ cells/peptide pool) were stimulated for 6 h in the presence of 2 μg of anti-CD28 and anti-CD49d MAbs/ml (BD PharMingen, http://www.pharmingen.com). The last 5 h of stimulation occurred in the presence of 10 μg/ml brefeldin A (Sigma, http://www.sigmaaldrich.com), with no stimulation as the background control or with phorbol myristate acetate (PMA) as the positive control, or peptide pools having the same amino acid sequences as GFP, or Ebola GP protein as the negative control (Figures S2 and S3). All peptides used in this report were 15 mers overlapping by 11 amino acids that spanned the complete sequence of the protein. Cells were permeabilized and fixed with Cytofix/Cytoperm and stained with monoclonal antibodies (rat anti-mouse cell surface antigens CD3-PE, CD4-PerCP and CD8-APC; BD PharMingen) followed by multiparametric flow cytometry to detect the IFN-γ (IFN-γ-FITC) and TNF-α (TNF-α-FITC) –positive cells in the CD4^+^ or CD8^+^ T-cell population. Statistical analyses in observed CD4^+^ and CD8^+^ responses between control-vaccinated and test article–vaccinated mice were performed by the *t*-test using Microsoft Excel software (http://www.microsoft.com).

### Flow Cytometry

Samples were assayed on an FACSCalibur instrument using CELLQuest software (BD Biosciences, http://www.bdbiosciences.com). The collected data were analyzed with FlowJo 6.1 software (Tree Star, http://www.flowjo.com).

### ELISA

ELISA plates of 96 wells were coated with 100 μl/well purified GFP (BD Biosciences) at 2.5 μg/ml and kept overnight at 4 °C. The GFP was removed, and each well was blocked with 200 μl of PBS containing 10% FBS for 2 h at room temperature. The plates were washed twice with PBS containing 0.2% Tween-20 (PBS-T). Then, 100 μl of serum from vaccinated mice was added to each well at a dilution of 1:100. The plates were incubated for 1 h at room temperature and washed. Afterward, 100 μl of horseradish peroxidase–conjugated goat anti-mouse IgG was added to each well. The plates were again incubated for 1 h at room temperature and washed. Subsequently, 50 μl of substrate (fast *o*-phenylenediamine dihydrochloride; Sigma) was added to each well. The plates were then incubated for 30 min at room temperature. The reaction was stopped by the addition of 100 μl of 1(N) H_2_SO_4_, and the optical density was read at 450 nm.

### Neutralization of Adenovectors with Human Sera

One hundred samples of human sera from volunteers enrolled in VRC-sponsored HIV trials in the United States were obtained from the VRC Immunology Core Laboratory. The sera were diluted with Dulbecco's modified Eagle's medium (1:12) and mixed with the indicated rAd vector encoding GFP for 1 h at room temperature. The neutralized virus was used to infect 293 cells at 500 PU/cell for 2 h, and GFP expression was analyzed by flow cytometry at 24 h post transduction.

### Pyrogenicity Assay

The 11- to 16-wk-old New Zealand White rabbits were administered 10^12^ particles of vector intramuscularly. This dose ensured that 100% of the animals injected with WT capsid rAd5 would have elevated body temperature because 10^11^ particles induced fevers in 60% of the animals (unpublished data). Body temperature was measured using subcutaneously implanted thermometers (BMDS transponder IPTT-200; BioMedic Data Systems Inc, http://www.bmds.com) at the nape of the neck.

## Results

### Transduction of Different Cell Types by WT and CAR^−^RGD^−^ Ad5 Vectors

**Figure 1:**
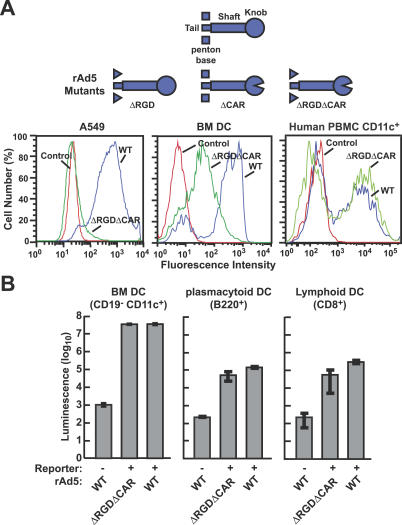
Specificity of WT or CAR^−^RGD^−^ rAd Vectors for Different Cell Types: Mutant Virus Retains Ability to Infect DCs but Not Other Cell Types (A)****GFP expression following transduction by rAd5 vectors with WT capsid proteins or mutated fiber and penton base proteins at 1,000 viral particles per cell. Transduction of human squamous epithelial cell A549, mouse BM-derived DCs, and human CD11c^+^ PBMCs was assessed by flow cytometry at 48 h post transduction. (B) Transduction by the indicated rAd vectors expressing luciferase was evaluated in mouse DCs. BM cells were isolated from BALB/c mice and cultured for 1 wk in the presence of GM-CSF [19]. The cells were transduced with indicated viruses at 3,000 viral particles per cell, and at 24 h postinfection, cells were stained with anti-CD19 and -CD11c antibodies and sorted into CD19^−^ and CD11c^+^ cells with FACSDiVa (BD Biosciences). Mouse plasmacytoid and lymphoid DCs were isolated with magnetic cell sorting and transduced with the indicated viruses at 10,000 viral particles per cell. Luciferase expression was measured at 24 h after transduction. The average of three infections is shown, along with standard deviations.

**Figure 2:**
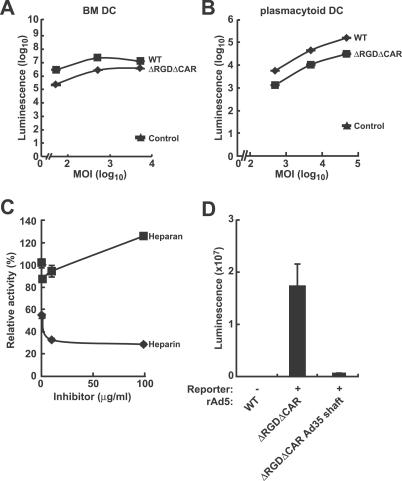
Transduction Efficiencies of WT and Mutant rAd Vectors in DCs and Sensitivity of Mutant Adenoviral Vector Infectivity to Heparin Inhibition Murine BM DCs (A) or human plasmacytoid DCs (B) isolated from PBMCs using MACS magnetic cell separation (Miltenyi Biotech Inc) were transfected with the indicated viral vectors expressing luciferase and luciferase expression were analyzed at 24 h postinfection. The control consisted of cells mock-transfected with culture medium. The data are the representatives of three independent transfections. (C) Murine BM DCs were preincubated with heparin or heparan sulfate for 30 min at room temperature and then transfected with the CAR^−^RGD^−^ rAd at 100 viral particles per cell, and relative luciferase activity was evaluated in a dose-response fashion to heparin or heparan sulfate at 24 h postinfection. The data shown are representative of three independent experiments. (D) BM-derived DCs were transduced with the CAR^−^RGD^−^ rAd or CAR^−^RGD^−^ rAd with Ad35 shaft at 3,000 viral particles per cell, and luciferase expression was analyzed at 24 h postinfection.

To identify the viral component responsible for gene transfer into specific cell types, mutant adenoviral vectors were constructed and tested in vitro for gene transfer and expression. The CAR^−^RGD^−^ rAd vector failed to mediate gene transfer into a number of epithelial and fibroblast cell lines of human or mouse origin that were readily transduced with a similar amount of WT rAd expressing a GFP reporter (Figures 1A, left, and S1). The specificity of CAR^−^RGD^−^ rAd was evaluated further in DC subsets and mononuclear cells derived from alternative tissues. Murine BM cells were isolated and incubated with mGM-CSF to promote differentiation into BM DCs [19]. The CAR^−^RGD^−^ rAd readily transduced these cells as measured with a GFP reporter, although transduction of this mixed population was more efficient with WT virus (Figure 1A, center). Unseparated human CD11c^+^ mononuclear cells derived from peripheral blood were also transduced by the mutants with similar efficiency to the WT virus (Figure 1A, right). When these cells were purified to yield murine BM DCs by sorting CD19^−^CD11c^high^ cells, the CAR^−^RGD^−^ rAd vector transduced these cells with similar efficiency to the WT virus, as determined with a luciferase reporter (Figure 1B, left). The CAR^−^RGD^−^ rAd vector was able to transduce other DC subsets from alternative tissues: both mouse B220^+^ (plasmacytoid) DCs and CD8^+^ (lymphoid) DCs derived from spleen cells were readily transduced by the mutant virus (Figure 1B, middle, right). A titration of input vector showed slightly lower transduction efficiencies by the CAR^−^RGD^−^ rAd vector, as measured by slightly reduced luciferase reporter activity in murine BM-derived and plasmacytoid DCs; nonetheless, the transduction was comparable to the WT capsid vector over a 2-log range of multiplicities of infection (Figure 2A and 2B). These findings indicate that transduction of several DC and mononuclear subsets is independent of CAR and integrin binding.

Adenoviral infection mediated by the shaft region of the fiber protein has been reported for certain cell types. Fiber shaft structure is specific to different serotypes, with respect to both the specific amino acid sequence and shaft length as measured by the number of repeats in each fiber. A KKTK motif has been identified in the Ad5 shaft that mediates transduction of some cells, and transduction mediated by this motif is sensitive to inhibition by heparin [20–22]. To test the role of the KKTK motif, we evaluated transduction of DCs in the presence of heparin sulfate or heparan sulfate, an alternative proteoglycan that served as a negative control. Heparin sulfate nearly completely inhibited CAR^−^RGD^−^ rAd gene transfer to murine BM-derived DCs, in contrast to heparan sulfate (Figure 2C), which suggests that ionic interactions contained within the fiber shaft are necessary. We also substituted the Ad5 shaft with the Ad35 shaft, which lacks the KKTK motif, in the CAR^−^RGD^−^ mutant. Gene transfer to murine BM-derived DCs by this mutant was substantially reduced (Figure 2D).

### Immunogenicity of Mutated Ad5 Vectors in Mice

**Figure 3:**
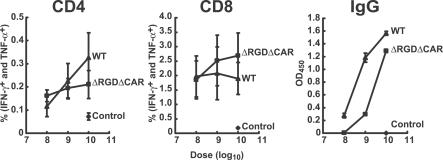
Immunogenicity of WT and Mutant rAd Vectors Dose-response analysis of WT or CAR^−^RGD^−^ mutant rAd immunogenicity in vivo. Cellular (CD4, CD8) and humoral (IgG) immune function was analyzed at the indicated doses of rAd vector. Spleen cells were stimulated with GFP peptides or Ebola virus glycoprotein (EB peptides) as the negative control, or peptide dissolving solvent as background control, and stained for the indicated cell surface markers and intracellular cytokines using fluorochrome-conjugated monoclonal antibodies. The percentage of intracellular IFN-γ^+^ (IFN-γ-FITC) and TNF-α^+^ cells in total CD4^+^ or CD8^+^ in GFP peptide-stimulated samples was subtracted from the background control samples (no peptide stimulation). The samples were also stimulated with an irrelevant peptide pool derived from Ebola virus glycoprotein (Figure S2). The average of data from five mice in each group and the standard deviations are shown. For CD4^+^ and CD8^+^ responses, at each dosage, there is no significant difference between WT and the mutant (*p* > 0.05, *t*-test by Microsoft Excel). Mouse sera were diluted at 1:100, and anti-GFP IgG was measured by ELISA. The averages of five mouse sera in each group with standard deviations are presented. Mutant generated less IgG response compared with WT at each dosage (*p* < 0.05, *t*-test by Microsoft Excel).

**Figure 4:**
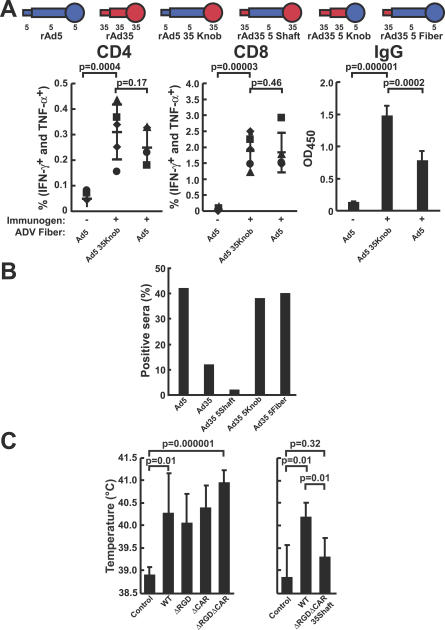
Ad5 Shaft Does Not Contain Human Neutralization Epitopes and Contributes to In Vivo Immunogenicity and Pyrogenicity of rAd5 Vector (A) Mice were injected with rAd5 or a chimeric rAd5 containing the knob region from Ad35 at 10^10^ viral particles (Ad5 35knob). Cellular immune responses were assessed by analysis of intracellular cytokine staining in CD4^+^ or CD8^+^ cells, and antibody responses were assessed by ELISA in the same animals (*n* = 5). The samples were also stimulated with an irrelevant peptide pool derived from Ebola virus glycoprotein (Figure S3). (B) Neutralization of rAd vectors with 100 individual human sera. Recombinant Ad vectors with chimeric rAd5-rAd35 fiber proteins were incubated with serum diluted 1:12 for 1 h and added to the cells, and the GFP fluorescence intensity arising from infection was determined by flow cytometry. Sera that inhibited GFP expression by more than 50% were considered positive for neutralization. (C) Pyrogenicity of rAd5 vectors evaluated in rabbits. Rabbits were injected intramuscularly with 10^12^ particles of the indicated rAd5-luciferase vectors, and body temperature was measured at 24 h postinjection for each animal (*n* = 5 for all the groups except *n* = 3 in the control group of the right graph). The control groups were injected with the storage and dilution buffer for rAd vectors. The rAd5 vectors in the left panel had WT Ad5 capsid proteins (WT), a deletion of the RGD motif in the penton base protein (ΔRGD), a mutation in the fiber knob that prevented CAR binding (ΔCAR), or both capsid protein mutations (ΔRGDΔCAR). The two rAd5 vectors in the right panel were the WT capsid vector (WT) and a ΔRGDΔCAR vector with the Ad5 shaft region replaced with the Ad35 shaft region (ΔRGDΔCAR 35shaft).

To define the Ad5 viral determinants of immunogenicity in vivo, mice were injected with increasing amounts of different recombinant viruses expressing GFP as the antigen. When the WT and CAR^−^RGD^−^ rAd were analyzed, there was no significant difference (*p* > 0.05) in the peak levels of cellular immune response elicited by the WT and double-mutant vectors (Figure 3). Thus, although the specificity of the CAR^−^RGD^−^ adenoviral vector differs markedly from the WT vector, its ability to bind to DCs remains unchanged, and it is potently immunogenic in vivo. Finally, to confirm the role of the shaft in stimulating immune responses by rAd vectors, the immunogenicity of rAd5 was compared to rAd5 with Ad35 knob transposed onto the fiber. Both vectors stimulated responses that were significantly above those from a vector containing a control insert (Figure 4A). Instead of binding to CAR, the Ad35 knob normally binds to CD46 in humans, but CD46 is absent from nearly all mouse tissues [23]. Consequently, the rAd5 vector containing the Ad35 knob is expected to have little cellular binding dependent on the knob. However, both vectors can utilize the Ad5 shaft and showed comparable CD4 and CD8 intracellular cytokine staining and increased antibody titers (Figure 4A), confirming the role of the Ad5 shaft independent of the Ad5 knob in T-cell immunogenicity.

### Neutralization of WT and Chimeric Ad5 and Ad35 Vectors by Human Sera

Because the Ad5 shaft had a significant role in the adaptive immune response, it was possible that the shaft was a target for neutralizing antibody. A panel of rAd35 vectors engineered with Ad5 fiber domains were compared to rAd5 for susceptibility to neutralization by human serum samples obtained from healthy adults. Transposition of the Ad5 shaft to rAd35 did not affect neutralization; however, the presence of the Ad5 knob alone changed the neutralization profile of rAd35 to that of rAd5 (Figure 4B). Thus, the shaft region did not contain human neutralization epitopes.

### Pyrogenicity of Ad5-Based Vectors in a Rabbit Model

To determine whether the Ad5 shaft contributed to the pyrogenicity observed after the administration of rAd vectors, rabbits were injected with a high dose, 10^12^ particles, of rAd5 vectors. All vectors containing the rAd5 shaft, including those with mutations in the penton base RGD and fiber CAR-binding domain, induced a statistically significant increase in body temperature (Figure 4C, left), including those in which both the CAR and integrin interactions had been ablated. In contrast, the group of animals that received the CAR^−^RGD^−^ rAd5 vector without the Ad5 shaft had significantly lower mean body temperature relative to the group with the Ad5 shaft (Figure 4C, right). Thus, the ability of a vector to induce a potent pyrogenic response was also associated with the Ad5 shaft, consistent with the observation that the Ad5 shaft is associated with transduction of DCs and stimulation of adaptive immunity.

## Discussion

In this paper, we have evaluated the contribution of the known receptors of adenoviral vectors as vaccines. Previous studies have shown that the CAR-binding region of the adenoviral knob and the integrin-binding motif RGD in the penton base protein are responsible for targeting the adenovirus to a variety of cell and tissue types in vivo [7,12,24–26]. In skeletal muscle, the CAR^−^RGD^−^ rAd was previously shown to transduce 100-fold less efficiently compared to a WT capsid vector [14]. The present study evaluates the contribution of these receptors to adenovirus infectivity and immunogenicity. Mutations in the CAR- and integrin-binding domains were not required for immunogenicity, despite their significant effect on targeting of the virus to many cell types including skeletal muscle. The CAR^−^RGD^−^ vector was clearly still competent for transduction, but the cell types transduced following an intramuscular administration must have been a subset of those transduced by the WT capsid vector. It has been previously suggested that DCs likely play a critical role in the ability of the adenovirus to elicit immunity in vivo [27,28], but the mechanism by which rAd5 targets these cells was unknown. It has been suggested that immature cells are infected by the virus, leading to differentiation to more mature DCs that may more effectively present antigen in vivo [29–33]. In vitro, adenovirus can induce maturation of BM DCs, and the penton RGD has been shown to be involved in such stimulation [31,34]. Our data suggest that adenovirus can utilize pathways other than the RGD–integrin interaction to mature DCs in vivo, as deletion of the penton RGD has no effect on immunogenicity of the vector. An alternative proposal is that adenovirus targets more mature DCs more effectively in vivo. Because of the multiple cell-binding specificities of adenoviral vectors, the relative contributions of these determinants to immunogenicity and pyrogenic toxicity provide important information relevant to vaccine design for gene-based and other modes of antigen delivery in vivo. This study suggests that the shaft contributes to the targeting of the adenovirus to DCs, which likely mediate antigen presentation and enhance immune reactivity in vivo. Within the shaft sequence is a repetitive heparin-binding motif, KKTK, that may mediate this effect. The shaft domain has also been implicated in Ad5 targeting to hepatic cells and cytokine release when administered through intravenous injection in mice [15,16]; however, its relation to the pyrogenic response could not be defined in this model. Although CAR^−^RGD^−^ virus can bind to DCs and gene transfer is blocked by heparin, but not heparan sulfate, it is not clear that the KKTK domain alone mediates interaction with DCs; nonetheless, it is clear that the shaft domain is involved and that this interaction is dependent on a heparin-like receptor interaction. This finding suggests that modifications of the adenoviral fiber may allow design of targeted adenovirus vectors modified to avoid toxicity and reactogenicity. Fever that was observed as a component of a serious adverse event in a human gene therapy trial [6] after intraportal artery infusion of high doses of rAd5 could possibly have been mediated by fiber interactions. Selective modification of the shaft region, for example, by substitution with the Ad35 shaft (which does not have putative heparin sulfate proteoglycan-binding motifs), as shown here, may assist in avoiding this complication. In addition, the immune response to vector capsid proteins has limited the repetitive use of adenoviral vectors for vaccine-induced immune responses. The ability to define specific motifs within the adenoviral fiber that facilitates DC binding and entry may assist in the development of synthetic vectors that target these cell types specifically. Interestingly, it has been difficult to identify antibodies directed to the shaft region of adenoviral vectors (Figure 4B), raising the possibility that this motif may be protected from immune recognition and could be retained in chimeric rAd from different serotypes or with synthetic vectors. This knowledge may also assist in the design of Ad vectors that could target DCs by other receptors that may not lead to the pyrogenic response. For example, DC-specific ligands, such as DC-SIGN or Langrin, can be incorporated into the detargeted CAR^−^RGD^−^ vector with the shaft region from Ad35 that does not cause a pyrogenic response, to build alternative DC-targeted adenovirus vectors. These studies therefore lend insight into the mechanisms of adenoviral immune targeting at the same time they suggest possible means for targeting specific cells in vivo for future gene-based vaccines.

## Supporting Information

Figure S1 Ability of WT or CAR^−^RGD^−^ rAd Vectors to Infect Alternative Cell Lines The indicated WT and mutant fiber and penton rAd vectors expressing GFP were evaluated as in Figure 1. Transduction of murine NIH 3T3 fibroblasts (10,000 viral particles per cell), Hepa1–6 human hepatocellular carcinoma (500 viral particles per cell), MEL-12 mouse erythroleukemia (2,000 viral particles per cell), and MM-14 mouse multiple myeloma cells (2,000 viral particles per cell) were analyzed by flow cytometry.
(1.5 MB EPS)

Figure S2 Cellular Response to Irrelevant Peptide Pool Derived from Ebola Viral Glycoprotein Spleen cells were stimulated with peptide derived from Ebola viral glycoprotein, and T-cell response was measured as shown in Figure 3.
(426 KB EPS)

Figure S3 Cellular Response to Irrelevant Peptide Pool Derived from Ebola Viral Glycoprotein Spleen cells were stimulated with peptide derived from Ebola viral glycoprotein, and T-cell response was measured as shown in Figure 4A.
(388 KB EPS)

## Abbreviations

BM: bone marrow
CAR: coxsackievirus and adenovirus receptor
DC: dendritic cell
GFP: green fluorescent protein
PU: particle unit
rAd: recombinant adenovirus
rAd5: recombinant adenovirus serotype 5
VRC: Vaccine Research Center
WT: wild-type
